# Some Overlooked Points in Liver Troubles

**Published:** 1897-07

**Authors:** Jacob Michaux

**Affiliations:** Richmond, Va., Professor of Obstetrics, University College of Medicine, Richmond, Va.


For the Texas Medical Journal.

### SOME OVERLOOKED POINTS IN LIVER TROUBLES.

BY JACOB MICHAUX, M. D., RICHMOND, VA.,
Professor of Obstetrics, University College of Medicine, Richmond, Va.

[Read before Richmond Academy of Medicine and Surgery, May 25, 1897.]

THE above caption opens far too wide a subject to be dealt with exhaustively, except in a systematic treatise; hence no attempt will be made to cover the whole field. My object is to give my personal experience and observations, improved by all the extraneous sources I could command in the short time at my disposal.

First, I desire to direct your attention to the confusion that exists regarding the two widely different and entirely distinct conditions known as *jaundice* and *biliousness.* A proper conception of the difference between these conditions is most important, because of the *radically different treatment* required for them. Furthermore, it is important on account of the aid to diagnosis of a just appreciation of the two conditions.

The occurrence of jaundice is, as a rule, only possible when the *common* duct is obstructed and the *secreted bile is reabsorbed.*

[There are one or two exceptions, viz: A rare form of blood dyscrasia and yellow fever.] Thus it appears that jaundice is the result of the absorption of bile; whilst biliousness is the *muddy*, *dirty*, *discoloration* resulting from the non-elimination of the elements which constitute the bile. Chemistry proves that color depends upon the arrangement of atoms; hence it is easily seen that the characteristic color of the bile could not be found in the tissue before the bile is elaborated from the crude materials in the blood. Observation and experience have shown this view to be correct. *Biliousness*, therefore, is only the result of functional derangement of the liver; whilst *jaundice* is the result of obstruction of common duct (from any of several causes, such as catarrh, tumor, parasite, or gall-stone, etc.) In the latter condition the liver is often perfectly normal; but is unable to deliver its secretion to the intestine.

It is readily seen from the foregoing statements that the treatment of the two conditions must be radically different. It is manifestly bad therapy to stimulate the liver in jaundice, for the reason that the organ is not at fault, and an increase of the secretions would endanger the integrity of the tubes and increase the pain and pressure. Better far to give Belladonna in full doses to dilate the tube by its action on unstripped muscular fibre, and soda bicarbonate to render the bile more fluid, thus to facilitate its outflow; the salines to deplete and relieve pressure.

In the case of biliousness we simply stimulate the functions of the liver, and for this we have many efficient remedies.

Among these are some of great value too little used, viz.: ipecac and euonymus. These, in combination with calomel, I think, are the best. I am aware of the mooted question with regard to the action of calomel; but I must confess that my faith in it as a cholagogue remains unshaken.

DISCUSSION.

Dr. Landon B. Edwards said there had long been a recognized difference between jaundice and biliousness. Jaundice rarely produces material headache, and is not often connected with diseases of the liver itself. In short, jaundice is a sign or symptom of obstruction of the bile-ducts—whether due to catarrh of the duodenum or to the pressure of tumors or distended organs upon the bile ducts. In other words, "*yellow* jaundice," as people usually style it, recognizes that bile had been formed, but some obstruction along the course of the ducts prevents its excretion into the alimentary canal. The formed bile is dammed back first into the liver cells and then into the general circulation; in short, it is absorbed into the system. Circulating in the blood, it stains every tissue of the body and seeks elimination by the kidneys, causing what is known as mahogany urine. *Hæmatogeneous* jaundice, on the other hand, refers to the retention in the blood of materials which were intended to make up the bile. In other words, it is "embryonic" bile—if such a term may be allowed—in the blood; it is not formed bile. The appearance of this hæmatogenous jaundice reminds one of that muddy sallow color recognized as a malarial complexion, or even as a cancerous cachexia, rather than of the pumpkin yellow jaundice.

As to biliousness—we all recognize its occurrence, but do not understand its pathology. It is often recognized as a bilious sick headache. Sometimes it is marked by bilious vomiting; at other times it is constipation, and sometimes there may be the absence of the usual marks of bile in the bowel discharges, but there is not the pumpkin yellow jaundice. The appearance partakes rather of the nature of hæmatogenous than hepatogenous jaundice. Apparently biliousness is due to inactive liver-cell action, but the condition is not understood. *As to treatment of jaundice*, due simply to catarrh of the duodenum or bile-ducts, experience had taught him the value of calomel and soda. He has no theory to offer in explanation of the action, for when one undertakes to reason out the *modus operandi*, he is confronted with questions which he can not answer. He simply knows it does good. This treatment is also useful in biliousness. Non-emetic or diaphoretic doses of ipecac are valuable adjuvants. Nitro-glycerine and Nitrite of amyl also help. In chronic conditions, phosphate of soda is beneficial. Where there appears to be impaction of the common or hepatic ducts by bile or gall-stones, he thinks he has seen beneficial results from copious draughts of olive oil. Some of the younger generation of therapeutists ridicule the supposed action of olive oil because they cannot explain it. Perhaps some of the copious draughts of oil finds its way along the walls of the common duct, and even around the obstructing gall-stone, thus lubricating the channel for its extrusion. If the stone should be too large to be moved by natural process, then the surgeon will have to be called to the help of the physician.

Dr. J. S. Wellford was interested in the jaundice of the newborn. The child, when born, was red, and went gradually to white, but juandice nearly alway was present. He recognized the fact that it was somewhat due to the fœtal anatomy of the liver, which before birth discharged some of the functions of the lungs, and he was also aware of the condition, jaundice neonatorum, but he was somewhat at a loss in some cases.

A child born at the eighth month of uterine life was badly developed, weighing two or three pounds, and had an apparent absence of the fontanelles, which, however, developed in two or three days.

If there was no discoloration of the conjunctiva, he regarded the condition as normal catarrh, but, in this instance, the jaundice persisted for nearly a month.

Some years ago, he had an attack of acute jaundice, well-marked and painful. It was treated most actively with saline aperients, till he had six operations daily, and he noticed that, after each operation, the skin assumed a lighter hue.

The question of biliousness can be satisfactorily explained in a number of instances: the liver was not performing its functions, *e. g.*, making bile, and the ptomaines, instead of being eliminated, were absorbed, producing certain symptoms, among them a muddy, but not jaundiced, condition of the skin. It was a lack of elimination, seen whenever the liver was involed. In malarial regions, there are always liver complications, producing muddy complexion, etc.; and most cases of biliousness result from debauching, over-feeding, etc., and were generally relieved by saline aperients made alkaline.

Dr. Jacob Michaux, in closing the discussion, said his attention had been directed to the subject because of the confusion existing regarding terms and treatment, the former being distinctly important from both a diagnostic and therapeutic point of view. He approved everything brought out in the discussion, and reported the following case:

Woman, over 70 years of age, upon whom he operated for gall-stone about ten years ago, had pain and local indications of obstruction of the common duct, followed by jaundice, which became intense. The usual remedies were given, but without avail; the urine became of a mahogany color; stools were entirely devoid of bile, etc. She was taking very little food—not enough to sustain her, as milk and rice. An operation to relieve her was done, but, unfortunately, without result. The abdomen was opened and liver and gall-bladder exposed, but the endeavor to trace the duct was vain, because of the mass of inflammatory exudate which matted the omentum and intestines almost solidly, and he feared to use force. The gall-bladder was incised, and with a long, probe-pointed, flexible probe he thought he could follow up the tube and push out the stone, but could not. Then he endeavored to reach the duct through the intestine, and again failed. In the bladder was found a stone of considerable size. The edges of the gall-bladder were stitched to those of the abdominal wound, and two small drainage tubes of india rubber were inserted, and the abdominal wound closed. For several weeks the gall-bladder was washed out, but it was afterwards found unnecessary to do so, as the flow of bile through the drainage tubes was considerable, the amount reaching twelve ounces in twenty-four hours. As a result, the skin and urine cleared up, but the character of fæces remained unchanged and digestive disturbances continued.

Gaining consent for another operation, he performed one that, to his knowledge, had never been done before, and that had been merely suggested by reading Harley as to the establishment of a fistula. It was done in February, 1888. Openings were made in the gall-bladder and a knuckle of the duodenum, and the edges stitched together. The abdominal incision was closed. The patient died on the night following the operation, and he had no further opportunity for testing its efficiency.

A point of interest was the remarkable clearing of the skin and urine, which was absolute proof of the position taken with regard to the etiology of jaundice.

Regarding biliousness, it appeared to be due simply to non-elimination of the materials going to form bile.

